# Home Health Care Problem with Synchronization Visits and Considering Samples Transferring Time: A Case Study in Tehran, Iran

**DOI:** 10.3390/ijerph192215036

**Published:** 2022-11-15

**Authors:** Mahyar Mirabnejad, Hadi Mohammadi, Mehrdad Mirzabaghi, Amir Aghsami, Fariborz Jolai, Maziar Yazdani

**Affiliations:** 1 School of Industrial Engineering, College of Engineering, University of Tehran, Tehran 1439955961, Iran; 2 School of Industrial Engineering, K.N. Toosi University of Technology (KNTU), Tehran 1999143344, Iran; 3 Research Centre for Integrated Transport Innovation, School of Civil and Environmental Engineering, The University of New South Wales, Sydney 2052, Australia

**Keywords:** home health care, metaheuristics, samples transferring time, skill requirements, case study, transportation

## Abstract

Health care facilities have not increased in response to the growing population. Therefore, government and health agencies are constantly seeking cost-effective alternatives so they can provide effective health care to their constituents. Around the world, health care organizations provide home health care (HHC) services to patients, especially the elderly, as an efficient alternative to hospital care. In addition, recent pandemics have demonstrated the importance of home health care as a means of preventing infection. This study is the first to simultaneously take into account nurses’ working preferences and skill levels. Since transferring samples from the patient’s home to the laboratory may affect the test results, this study takes into account the time it takes to transfer samples. In order to solve large instances, two metaheuristic algorithms are proposed: Genetic Algorithms and Particle Swarm Optimization. Nurses are assigned tasks according to their time windows and the tasks’ time windows in a three-stage scheduling procedure. Using a case study set in Tehran, Iran, the proposed model is demonstrated. Even in emergencies, models can generate effective strategies. There are significant implications for health service management and health policymakers in countries where home health care services are receiving more attention. Furthermore, they contribute to the growing body of knowledge regarding health system strategies by providing new theoretical and practical insights.

## 1. Introduction

Many countries have experienced considerable economic pressure due to the rise in health care expenditures [1]. In Canada, for example, health care costs have exceeded GDP growth in the last 13 years [2]. The US health care system also faces similar challenges [3]. As a result of rising health care costs, even developing nations have been under considerable economic stress [4]. Governments and health agencies are therefore always looking for new and cost-effective ways to serve their constituents [5,6]. Home health care (HHC) services are an integral part of many health care organizations around the world, helping patients, especially the elderly, recover from hospitalization or remain at home safely without unnecessary hospitalization as a crucial sector [7,8,9]. However, these strategies for providing health services are always associated with difficulties, particularly in terms of logistics and transport [10,11].

Health care can be provided to patients at home; however, the transportation costs and travel distances need to be decreased in order to further improve the efficiency of HHC services. An HHC system typically consists of caregivers, patients, and their requests for medical services [12,13,14,15]. Assigning caregivers to these services results in the creation of their travelling routes. As a result, Vehicle Routing Problems (VRP) and Nurse Rostering Problems (NRPs), both well-known Operations Research problems, are the foundations of Home Health Care Routing-Scheduling Problems (HHCRSPs) [16]. Nurses are dispatched by service providers on specific days during specific times. Other aspects of the problem that need to be considered are nurses’ abilities, patients’ preferences, and disease types, as well as sending nurses based on the patient’s condition. Moreover, these organizations may provide home medical tests, send samples to laboratories, and send medicine. Additionally, the COVID-19 pandemic has highlighted the importance of HHC by increasing the demand for hospital services and redirecting medical, health and capacity resources nationwide [17,18,19]. The importance of HHC becomes more important when it comes to preventing infection among children and the elderly due to their increased vulnerability [20].

This study highlights some new features of the HHC problem for the first time. As a result of this study, the following contributions are made: (1) nurses’ preferences and their degree of skill are taken into consideration when synchronized visits take place. (2) Since delays in transporting some samples to the laboratory can affect test results, sample transfer times from the patient’s home to the laboratory are taken into account. (3) In order to assign nurses to tasks, a new three-stage scheduling procedure has been developed. Moreover, due to the problem’s high complexity, solving these problems in a reasonable amount of time becomes exceptionally difficult. In many instances involving large-scale problems, exact methods or commercial solvers cannot handle the problem’s complexity and cannot provide desirable solutions in a reasonable amount of time. A comprehensive review of HHC papers was published in [12,21,22], which demonstrates that this aspect of the problem has received considerable attention; however, this research direction is still an open-ended research area avenue. In solving complex problems, the three most common types of algorithms are conventional algorithms, heuristic algorithms, and meta-heuristic algorithms. Although mathematical programming techniques have been widely employed [23], they are frequently inappropriate and ineffective when applied to large-scale problems. In lieu of these, heuristic algorithms are utilized. By replacing conventional algorithms, heuristic algorithms can save a great deal of computation time, but they may get stuck in local optima and fail to provide the optimal solution to a planning or scheduling problem. Numerous researchers have an interest in meta-heuristic algorithms due to their effectiveness and applicability in solving real-world problems [24,25,26,27]. Therefore, they have been used in many different research areas [28,29,30,31,32,33,34,35,36,37,38]. This paper utilizes GA and PSO, which have been extensively used in literature reviews for various types of vehicle routing problems [39], and they have demonstrated their capability in handling complex problems [40,41]. Some of the questions that will be answered in this research are:

(1) What is the effect of sample-related constraints?
(2) What is the relationship between nurses’ total working time and the number of nurses used?

To answer these questions and, also, to consider the features and constraints mentioned above, a mathematical model of the problem is presented and solved with the help of the exact and metaheuristic methods. Some characteristics of the problem have been obtained based on a service provider’s information in Tehran, Iran.

As a result, the paper is organized as follows: Section 2 reviews recent papers on this topic. In Section 3, the proposed model will be described, and the parameters, decision variables, and related mathematical models will be presented. The proposed algorithm is described in Section 4, and then, the computational results are presented om Section 5. The sensitivity analysis will be performed in Section 6. Finally, Section 7 and Section 8 present managerial insights, conclusions, and suggestions for future research.

## 2. Literature Review

Cheng and Rich [42] probably proposed the first mathematical model of HHC. The problem was modelled as a Vehicle Routing Problem with Time Windows (VRPTW) in which nurses were divided into full-time and part-time categories. Minimizing full-time nurses’ overtime, as well as part-time nurses’ work hours was the objective function. A two-stage algorithm was used to solve the problem. Bertels and Fahle [43] considered time windows to be either hard or soft. The latter type can be violated by considering a penalty in the objective function. A number of factors were taken into account, including patients’ preferences for nurses, nurses’ preferences, nurses’ qualifications to perform tasks, and the distribution of hard work among nurses. A Variable Neighbourhood Search (VNS) was used by Trautsamwieser and Hirsch [44] in solving problems related to 140 tasks and 13 nurses in an urban area and 512 tasks and 75 nurses in a rural area. There were seven terms in the objective function, all related to time characteristics. Comparing the results with actual assignments showed a 45% improvement in travel time. Trautsamwieser et al. [45] also considered the effects of natural disasters such as floods and earthquakes. The VNS method was used to solve real-life instances. Bard et al. [46] divided patients into two categories according to their time window: fixed and flexible. A fixed time window applies to the first category, while the second category can be viewed all day long, although the day on which the second category can be viewed is not determined. In addition, it was considered that a fully licensed therapist should visit patients on their first visit. Liu et al. [47] proposed a mathematical model with three indexes in which some clients may not be visited, so a penalty was added to the objective function. In the model, lunch break was considered for nurses at patients’ homes. The branch and bound (B&B) algorithm was used to solve the model, and tabu search and label correction algorithms were also used to solve the subproblems. As Rest and Hirsch [48] assumed nurses used public transportation, travelling times between patients can be affected by traffic and rush hour, depending on the nurse’s movement time between patients. Dynamic programming was used to calculate the travel time. For solving a single-day problem with uncertain service times, Yuan et al. [49] proposed a branch-and-price algorithm. By assigning a dummy demand and capacity to clients and nurses, a new formulation was developed to describe nurses’ qualifications. As nurses use different vehicles for displacement, Hiermann et al. [50] assumed that the time it takes to travel between two places depends on the type of vehicle. In order to solve the problem, a two-stage approach was used. In the first stage, constraint programming or random procedures were used to provide exact solutions, and in the second stage, VNS, memetic algorithms, scatter searches, or simulated annealing hyper-heuristics were used to improve the initial solutions. Wirnitzer et al. [51] presented a model that considers five different measures of continuity of care when considering a problem. On the basis of these five measures, five MIP models were introduced for monthly home health care. Mosquera et al. [52] introduced flexible task durations as their main contribution. As a result of this concept, a greater number of tasks can be accomplished more quickly. The time of each task was assumed to be limited with an upper and lower bound. A higher priority was given to tasks which duration could be reduced because of their medical nature. For HHC centres, Lin et al. [53] proposed two models that considered rostering, routing, and re-rostering simultaneously. In the first model, rostering and vehicle routing were incorporated simultaneously, while, in the second model, nurse re-rostering was considered because of sudden incidents (such as patient-specific visit times, nurse absences, etc.). Yuan et al. [54] considered that travel times and service times are stochastic. Minimizing the expected travel costs, considering the penalty for uncovered customers, and, also, unexpected failure costs were considered. In the case of a nurse arriving late to a patient’s home, the visit will be cancelled, and the nurse will go to the next patient. An approximation method was presented to obtain probability distributions for nurses’ arrival times.

There is a difference in the qualification level of nurses who are sent to patients’ homes. In addition to providing medical services, nurses provide psychological counselling and speech therapy, take samples of patients, and perform physiotherapy, as well as help with daily tasks, such as cleaning the house, taking a bath, and making meals. There are different approaches to considering this feature in the problem. In most papers similar to Mankowska et al. [55] and Carello and Lanzarone [56], the parameter was defined as 0 and 1 to consider qualification. The parameter is equal to 1 if the nurse qualifies to do the task; otherwise, it is 0. In Hiermann, Prandtstetter, Rendl, Puchinger, and Raidl [50] and Trautsamwieser and Hirsch [44], a number was allocated to nurse and patient as the qualification level. A nurse could visit a patient if the number assigned to him/her was equal to or greater than the number assigned to the patient. Fikar and Hirsch [57] considered a boundary for the differences between these two numbers. Khodabandeh et al. [58] developed a model for routing and scheduling nurses by downgrading the cost aspects of the problem by minimizing the difference between the nurses’ potential skills and their actual service plans. Another aspect of the problem is taking into account nurses’ and patients’ preferences. Nurses’ preferences include setting a time window for nurses’ availability, limiting working hours for nurses over a period of time, distributing tasks equally among nurses, rejecting patient visits, and limiting hard tasks.

Temporal dependencies between different tasks are another feature of the HHC problem. As an example, a patient should take medicine before eating at a specific time. Normally, a maximum and minimum time interval is considered between these two tasks. According to Mankowska, Meisel, and Bierwirth [55], some tasks have temporal dependencies (maximum and minimum durations). An example is taking medicine before or after eating a meal and for a minimum or maximum period of time. A new matrix presentation method was also provided. The rows of the matrix represent nurses, and the columns represent patients. Particularly in tasks with temporal dependencies, this method of presentation is appropriate. Based on constraints such as time dependence between tasks and nurses’ qualifications, Rasmussen et al. [59] modelled the problem as a set portioning problem. A branch and price method was presented to solve the model. The objective function was minimizing unvisited tasks (based on their priority) and total travel distance and also to fulfill nurses’ preferences. Two nurses must work simultaneously if these two numbers are equal to zero. For example, two people require two people to take a bath for a patient who is overweight or physically disabled. A generalized soft time window concept for synchronization constraints was developed by Rasmussen, Justesen, Dohn, and Larsen [59]. In the case of tasks that need two nurses simultaneously, there may exist a gap between the arrival time of the two nurses. According to the gap between the arrival times of the two nurses, a penalty was considered in the objective function. There is a detailed description of synchronization for VRP problems in [60].

Another aspect of HHC is the sampling and delivery of samples to laboratories [13,61,62,63]. The importance of drug distribution and medical equipment during COVID-19 has been discussed in many papers [64,65]. Shi, Boudouh, and Grunder [63] studied medicine delivery from depot to patients, sample collection from patients, and transportation from patients to labs based on the capacity and time window. It was considered that each patient’s medication dose was nondeterministic. Since each vehicle has a limited capacity, if the patient needs more medicine, the nurse returns to the depot, takes what is needed, and goes back to the patient’s house. In order to deal with uncertainty, a fuzzy chance-constrained programming approach was proposed. Goodarzian et al. [66] presented a bi-objective model minimizing service time and cost. A maximum working time per day was also considered to balance nurses’ working time. However, in this paper, the hospital and the laboratories were the starting and ending points. There was no consideration of sample delivery time to the laboratory. The problem was modelled as a simultaneous delivery and pickup time window vehicle routing problem by Liu, Xie, Augusto, and Rodriguez [61]. Various demand types were considered, including medicine transfer from the depot to the patient, special medicine transfer from the hospital to the patient, and transporting samples and unused medicine from the patient’s home to the medical lab and depot. In order to solve problems of large dimensions, GA and TS algorithms were presented due to the complexity of the presented model and the inability of Cplex to reach a solution in a reasonable amount of time. Liu, Xie, and Garaix [62] considered bringing medicine to patients’ homes and collecting samples from patients. However, dissimilar to a previous paper, the vehicles were assumed to be unlimited. Each patient’s visiting days were determined, and nurse routing and scheduling were determined for each day. Tabu search was used with feasible and unfeasible local searches to solve the problem. This method involves a vehicle picking up nurses and delivering them to their destination. Fathollahi-Fard et al. [67] proposed a supply chain for home health care that encompassed multi-objectives, multi-depots, and multi-periods. Their model considered the environmental impact and CO_2_ emissions. It was assumed by each patient that only one pharmacy should be assigned to them. To solve the problem, modified simulated annealing algorithms were proposed.

Uncertainty, as well as decision-making, regarding some policies is an inherent feature of problems taken from the real world. The problem discussed in this paper is not an exception to this rule. To make decisions about some policies in the field of HHC, it is necessary to evaluate these decisions in a process and then agree on them. In the process of reaching this consensus, it is necessary to coordinate between different points of view. In [68], a mathematical model was proposed to reach an agreement under conditions of uncertainty. In addition, in [69], mixed-integer robust maximum expert consensus models were proposed. To solve the proposed model, an improved Genetic Algorithm (GA) is proposed.

A summary of the main features of the problem was presented by the papers reviewed in the previous section. As a result, some aspects of the problem that have not been studied before were considered in this study. The following are some of these features:

- The delay in carrying some of the samples to the laboratory may affect the test results as well; therefore, in this paper, the time taken to transfer samples from the patient’s home to the laboratory is taken into consideration. The delivery of samples and medications has received less attention, as shown in Table 1.
- When two nurses are needed for a task at the same time, nurses’ preferences for working together are taken into account. Two nurses who work at a patient’s home for different reasons may not want to work together. Additionally, due to the greater coordination between two nurses, it may be wise to send two particular nurses for a task. This task is done according to the affinity matrix concept, which was referred to by Wang et al. [70]. An approach is provided for determining a threshold to avoid the model’s infeasibility due to this constraint. The paper mentioned above described this method in detail.
- It may be necessary to have two nurses with different degrees working simultaneously on tasks that require the presence of two nurses. For instance, if the presence of a nurse and a doctor is required simultaneously. In order to consider this situation, a number is assigned to nurses, along with the task that should be carried out by two nurses. Two nurses can complete that task if the sum of their assigned numbers is equal to the number that is assigned to the task.
- An integer programming model is presented in this paper for a home health care problem based on different features of the problem. Two metaheuristic algorithms are proposed for solving large instances: Genetic Algorithms and Particle Swarm Optimization.

In Table 1, the main characteristics of the problem are summarized.

**Table 1:** Constraints and assumptions considered in the related works.

Author	Nurse					Patient/Task				Others					
	Qualification	preferences	Time Windows	Breaks	Workload Balance	Temporal Dependencies	Preferences	Time windows	Continuity of Care	Multimodal	Period	Uncovered Tasks	Samples/Drugs Delivery	Data Set	Solution Procedure
Liu, Xie, Augusto, and Rodriguez [61]								√			Single		√	Benchmark	Genetic Algorithm/Tabu Search
Bard, Shao, and Jarrah [46]	√	√	√	√			√	√	√	√	Multi	√		Random data/Real-life data	Sequential greedy randomized adaptive search procedure
Mankowska, Meisel, and Bierwirth [55]	√					√	√	√			Single			Random generated	Adaptive Variable Neighbourhood Search
Braekers et al. [71]	√		√				√	√		√	Single			Real-life data	Large neighbourhood search
Liu, Yuan, and Jiang [47]	√		√	√				√			Single	√		Benchmark/Real life data	branch-and-price
Shi, Boudouh, and Grunder [63]								√			Single		√	Benchmark	Fuzzy credibility theory
Decerle et al. [72]	√		√			√		√			Single			Benchmark/Real life data	Memetic algorithm
Lin, Hung, Liu, and Tsai [53]	√	√			√		√				Multi			Real-life data	Harmony search algorithm
Parragh and Doerner [73]	√					√		√			Single			Benchmark	Adaptive Variable Neighbourhood Search
Yuan, Liu, and Jiang [54]								√			Single	√		Random data	Branch-and-price
Fathollahi-Fard, Govindan, Hajiaghaei-Keshteli, and Ahmadi [67]								√			Multi	√	√	Benchmark	Simulated annealing algorithms
Grenouilleau et al. [74]	√	√	√				√	√	√	√	Multi	√		Real-life data	Large neighbourhood search
Decerle et al. [75]	√	√	√		√	√	√	√	√	√	Multi	√		Benchmark	Memetic algorithm
Mosquera, Smet, and Berghe [52]	√	√	√				√	√	√	√	Multi			Real-life data	Local search algorithm
Decerle et al. [76]	√	√	√		√	√	√	√	√	√	Single			Benchmark	Memetic algorithm/Ant colony optimization
Fathollahi-Fard et al. [77]	√	√	√			√	√	√	√	√	Multi		√	Benchmark	Non-dominated sorting GA (NSGA-II), Adaptivememory SEO
This Paper	√	√	√			√	√	√			Single		√	A case study	Genetic Algorithm/Particle swarm optimization

## 3. Problem Statement

### 3.1. Assumptions and Problem Definition

Multiple nurses with varying qualifications work in patient homes under the HHC model. Depending on the patient’s condition, more than one task may be required on a given day. Each nurse should reach the final destination after performing a series of tasks. Nurses can start at different points. Additionally, each nurse’s final point and starting point can be different from another nurse’s. According to the formulation, all these points may coincide. Nurses are only available during certain hours. Throughout the day, each patient may have different requests. Each of these requests is considered a task. If there is no relation between them, they will be considered two completely separate tasks. There may also be temporal dependencies between tasks. $d_{ij}^{min}$ and $d_{ij}^{max}$ are the smallest and largest time distances between the start time of two tasks with temporal dependencies, respectively. In a situation that needs two nurses to do a task simultaneously, it will be $d_{ij}^{min}=d_{ij}^{max}=0$. It may be necessary for two nurses to be present at the same time, but their qualifications may differ. For tasks requiring the simultaneous presence of two nurses, each nurse is assigned a grade. The grade to each nurse is shown with $gr_{i}$ and related grade to tasks by $gr_{i}$. Nurses can be sent to do the same work simultaneously if the sum of their related grades is equal to the grade of the requested task. For tasks that require two nurses to be present simultaneously, nurses’ preferences are also taken into account. Some samples must be taken to laboratories during a set period of time, so there is an assumption that there is a contract with several laboratories in the city for transferring patients’ samples. The main variables of the problem are the starting time and waiting time. The objective function determines these variables in order to minimize the total amount of time nurses spend on work, which improves nurses’ satisfaction and reduces their costs. The notations are summarised in Table 2.

### 3.2. Notations

### 3.3. Mathematical Formulation

In this study, a new mathematical model for home health care is developed with the following aspects.

(1) $$Min\sum_{𝓸\in O\;,I\in L}\left({𝒮}_{1}-S_{0}\right)$$

(2) $$\sum_{𝒾\in J\cup O,𝓃\in N}X_{𝒾𝒿𝓃}=1\;\;\;\;\;\;\;\;\;\;\forall 𝒿\in J$$

(3) $$\sum_{𝓸\in O,𝒿\in J}X_{𝓸𝒿𝓃}\leq 1\;,\;\;\;\;\;\;\;\forall n\in N$$

(4) $$\sum_{𝒿\in J\;,\;\;I\in L}X_{𝒿𝓁𝓃}\leq 1,\;\;\forall 𝓃\in N$$

(5) $$\sum_{𝒾\in J\cup O\;,𝓃\in N}X_{𝒾𝒿𝓃}=\sum_{𝓀\in J\cup L,𝓃\in N}X_{𝒿𝓀𝓃},\;\forall 𝒿\in J$$

(6) $$\sum_{𝓀\in L\;𝒶\;𝒷\;,𝓃\in N}X_{𝒾𝓀𝓃}\geq \sum_{𝒿\in J\cup O,𝓃\in N}X_{𝒿𝓀𝓃},\;\forall 𝒿\in J^{𝓁𝒶𝒷}$$

(7) $$\begin{array}{c} \mu_{𝒾𝒿}\left(S_{𝒿}-S_{𝒾}\right)+\mu_{𝒿𝒾}\left(S_{𝒾}-S_{𝒿}\right)\\ \;\;\;\;\;\;\;\;\geq {𝒹}_{𝒾𝒿}^{𝓂𝒾𝓃}-M\left(2-\underset{𝓀\in J\cup O,𝓃\in N}{\sum }X_{𝒽𝒾𝓃}-\underset{𝒽\in J\cup O,𝓂\in N}{\sum }X_{𝓀𝒾𝓂}\right),\;\forall 𝒾,𝒿\\ \;\;\;\;\;\;\;\;\in J^{𝓅𝓇ℯ} \end{array}$$

(8) $$\mu_{𝒾𝒿}\left(S_{𝒿}-S_{𝒾}\right)+\mu_{𝒿𝒾}\left(S_{𝒾}-S_{𝒿}\right)\leq {𝒹}_{𝒾𝒿}^{𝓂𝒶𝓍}-M\left(2-\sum_{𝓀\in J\cup O,𝓃\in N}X_{𝒽𝒾𝓃}-\sum_{𝒽\in J\cup O,𝓂\in N}X_{𝓀𝒾𝓂}\right),\;\forall 𝒾.𝒿\in J^{𝓅𝓇ℯ}$$

(9) $$S_{𝒾}\geq S_{𝓀}-M(1-\sum_{𝓃\in N}X_{𝒾𝓀𝓃})-{𝓉}_{𝒾}^{𝓂𝒶𝓍}\sum_{𝓃\in N}X_{𝒾𝒽𝓃}\;,\forall 𝒾\in J^{𝓁𝒶𝒷},𝓀\in 𝓁𝒶𝒷$$

(10) $$S_{𝒿}\geq S_{𝒾}+w_{𝒿}+\left({𝒹}_{𝒾}+{𝓉}_{𝒾𝒿}\right)*\sum_{𝓃\in N}X_{𝒾𝒿𝓃})-M(1-\sum_{𝓃\in N}X_{𝒾𝒿𝓃}\;,\forall 𝒾\in J\cup O,\forall 𝒿\in J\cup L$$

(11) $$A_{𝒿}\leq S_{𝒿}\leq B_{𝒿},\forall 𝒿\in J\cup 𝓁𝒶𝒷$$

(12) $$S_{𝓸}\geq {𝒶}_{𝓸}*\underset{𝒿\in J,𝓃\in N}{\sum }𝓸𝒿𝓃,\forall 𝓸\in O\;$$

(13) $$s_{I}\leq {𝒷}_{I}*\sum_{𝒿\in J,𝓃\in N}X_{𝒾𝓁𝓃},\forall I\in L$$

(14) $$\sum_{𝓀\in J\cup O,𝓃\in N}{𝓍}_{𝓀𝒾𝓃}*ℊ{𝓇}_{𝓃}+\sum_{𝓀\in J\cup O,𝓂\in N}X_{𝓀𝒿𝓃}*ℊ{𝓇}_{𝓂}=ℊ{𝓇}_{𝒿}\;,\forall 𝒾,𝒿\in J^{𝓅𝓇ℯ};\mu \left(𝒾,𝒿\right)=1$$

(15) $$\underset{𝓀\in J\cup O}{\sum }{𝓍}_{𝓀𝒾𝓃}+\underset{𝓀\in J\cup O}{\sum }{𝓍}_{𝓀𝒿𝓂}-1\leq 𝓅{𝓇}_{𝓃𝓂},\forall 𝒾,𝒿\in J^{𝓅𝓇ℯ};\mu \left(𝒾,𝒿\right)=1,\forall 𝓃,𝓂\in N\;$$

(16) $$W_{𝒿}\leq W_{M𝒶X},\forall 𝒾\in J$$

(17) $$\sum_{𝒾\in J\cup O,𝒿\in J}{𝓍}_{𝒾𝒿𝓃}*{𝒹}_{𝒿}\leq D_{𝓂𝒶𝓍},\forall 𝓃\in N$$

(18) $${𝓍}_{𝒾𝒿𝓃\;}𝓂𝒶𝓍\left({𝓆}_{𝒾𝓃},{𝓆}_{𝒿𝓃}\right),\;\forall 𝒾,𝒿\in J\cup L𝒶𝒷\cup O\cup L,𝓃\in N$$

(19) $${𝓍}_{𝒾𝒿𝓃}\in \left\{0,1\right\},\forall 𝒾,𝒿\in J\cup L𝒶𝒷\cup O\cup L,𝓃\in N$$

(20) $${𝓌}_{𝒿\;}\geq 0,\;\forall 𝒿\in J\cup L𝒶𝒷\cup L$$

The objective function minimizes the total amount of time nurses spend working. A Constraint (2) ensures that all tasks are completed. According to the third and fourth constraints, each nurse must begin his/her work at the origin and then proceed to the final destination. Constraint (5) is the flow conservation constraint. Constraint (6) ensures that, if a nurse goes to a patient’s home to take a sample, she immediately goes to a lab. To prevent the nurse from taking samples to the homes of other patients, these constraints were put in place. In Constraints (7) and (8), there is a time relation between tasks in which there are temporary dependencies between them. As a result of Constraint (9), the sample will not be taken to the laboratory after the acceptable time has passed. Travelling time between the first and second tasks and waiting time for the second task are expressed in Constraint (10) as the relationship between the starting time of two consecutive tasks and the duration of the first task. Constraints (11)–(13) relate to nurses’ and patients’ time windows. Two nurses can be sent to a patient’s home simultaneously if the sum of their qualifications is equal to the degree assigned to the task. If two nurses want to work together, Constraint (15) allows them to do simultaneous tasks. The waiting time is limited by Constraint (16). Constraint (17) limits how much time each nurse spends on different patients. According to Constraint (18), a nurse will be assigned to a task if he/she is qualified to perform it, and the nurse’s and patient’s preferences should also be taken into consideration. The variable domains are defined by Constraints (19) and (20).

## 4. Solution Approach

Based on a real case, various examples were generated to analyze the presented model. Using GAMS, small examples were solved. It is always time-consuming to use exact methods [78,79,80]. As a result, meta-heuristic algorithms were required due to the high complexity of the problem. The proposed algorithms for this problem are explained and justified in the following.

### 4.1. Genetic Algorithm

**Figure 1:**
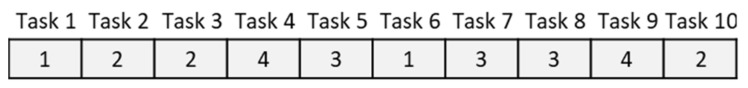
Solution representation.

There are many applications for Genetic Algorithms in routing and scheduling problems, e.g., [81,82]. There is also evidence that GA performs well in complex problems, particularly combinatorial optimization problems [39,83,84]. Some advantages of Genetic Algorithms are ease of use, the improvement of results over successive iterations, and the ability to escape local optima. It does not require any information about the function and the fact that it is suitable for large-scale optimization problems [85]. In Genetic Algorithms, points in the solution space are represented by chromosomes. In a meta-heuristic algorithm, one of the most important features is how solutions are presented. Genetic Algorithms are population-based; therefore, the way they display the solution should not take up more computer memory. In addition, each chromosome should correspond to a single point. Figure 1 shows a solution to a problem with 10 tasks and five nurses.

Decoding chromosomes and transforming them into one point of solution space is one of the most important aspects of showing the solution. Regarding assigning nurses to tasks, according to Figure 1, the assignment is as follows: nurse 1 to tasks 1 and 6; nurse 2 to tasks 2, 3, and 10; nurse 3 to tasks 5, 7, and 8; and nurse 4 to tasks 4 and 9. Additionally, in this chromosome, nurse 5 is unemployed. The arrangement of tasks that a nurse should do is specified according to the lower bound of the time window. For example, in the specified chromosome in Figure 1, the arrangement of tasks that should be done by nurse 3 will be 5, 8, and 7 if the lower bound of the time window in the specified task is $A\left(𝒿\right)=\left[20,\;10,\;50,\;30,\;40,\;80,\;90,\;70,\;60,\;100\right]$. Considering that there are different assumptions and constraints in the problem, the chromosomes and decoding process are designed so that some of the problem assumptions such as the establishment of the time windows lower bound for nurses and tasks and minimum time distance between two tasks that have precedence are always held. For other constraints of the problem, the repair and penalty mechanisms are used. This algorithm directs the related nurse to the nearest laboratory after completing each of these tasks. As a result, laboratories are not considered in the chromosome design, and the maximum amount of time a sample can spend in the laboratory is always considered. The three-stage decoding process of a chromosome is briefly described in this section:

1. Initial scheduling

Step 1: Sort all tasks according to their lower bound of the time window ($A\left(𝒿\right)$).

Step 2: In a sorted array, for each task = 1:$N_{𝒿}$ repeat step 3 (to calculate the arrival time of nurse *n* to task 𝒿,$𝓈\left(𝒿,𝓃\right)$ and step 4 (to calculate starting time of task 𝒿 by nurse 𝓃, $𝓉\left(𝒿,𝓃\right)$. In the following equations, $𝓉𝓉\left(𝒾,𝒿\right)$ is the travelling time from node 𝒾 to node 𝒿, $𝒹\left(𝒾\right)$ is the duration of task 𝒾, and $𝒶\left(𝓃\right)$ is the lower bound of the time windows for the nurse 𝓃. Additionally, $J^{Lab}$ is the set of tasks that requires sampling. 

Step3:

(21) $$𝓈(𝒿,𝓃)=\left\{\begin{array}{cc} Max\left\{A(𝒿),𝓉𝓉(𝓃,𝒿)+𝒶(𝓃)\right\},\;\;\;\;\;\; & if\;task\;𝒿\;is\;the\;first\;task\;of\;nurse\;𝓃\;and\;there\;is\;no\;prereuisite\;for\;task\;𝒿\\ Max\left\{A(𝒿),𝓉𝓉(𝓃,𝒿),\sum_{𝓃=1}^{N_{𝓃}}𝓉(𝒾,𝓃)+{𝒹}_{𝒾𝒿}^{min}\right\},\;\; & if\;task\;𝒿\;is\;the\;first\;task\;of\;nurse\;𝓃\;and\;task\;𝒾\;is\;prerequisite\;task\;𝒿\\ 𝓉\left(𝒾,𝓃\right)+𝒹\left(𝒾\right)+𝓉𝓉\left(𝒾,𝒿\right),\;\;\;\;\;\;\;\;\; & elseif(task\;𝒾\;was\;done\;before\;task\;𝒿\;by\;nurse\;𝓃\;and\;𝒾\notin J^{Lab}\\ 𝓉\left(𝒾,𝓃\right)+𝒹\left(𝒾\right)+𝓉𝓉\left(𝒾,Lab\right)+𝓉𝓉\left(Lab,𝒿\right),\;\;\;\; & elseif(task\;𝒾\;was\;done\;before\;task\;𝒿\;by\;nurse\;𝓃\;and\;𝒾\in J^{Lab} \end{array}\right.$$

Step 4:

(22) $$𝓉\left(𝒿,𝓃\right)=\left\{\begin{array}{cc} Max\left\{A\left(𝒿\right),𝓈\left(𝒿,𝓃\right)\right\} & if\;task\;𝒿\;do\;not\;have\;a\;prerequisite\\ Max\left\{A\left(𝒿\right),𝓈\left(𝒿,𝓃\right),\sum_{𝓃=1}^{N_{𝓃}}𝓉\left(𝒾,𝓃\right)+{𝒹}_{𝒾𝓃}^{min}\right\} & else\;\left(task\;𝒾\;is\;prerequisite\;task\;𝒿\right) \end{array}\right.$$

The arrival times of each nurse are calculated in step 3. It is calculated based on the dependency between the tasks, as well as their category. A starting time is also calculated in step 4. If the desired task does not have prerequisites, the starting time is determined according to the lower bound of the task’s time window and the arrival time of the nurse.

2. Rescheduling one

According to the above procedure, nurses’ departure times are as soon as possible, so waiting times are likely to increase. In order to correct the starting time of each nurse’s tour, the following procedures will be followed. In this regard, nurses are classified into two categories: dependent and independent. Nurses who are independent are those whose tasks do not require any prerequisites. The indicator (floating) for each independent nurse is defined as follows. Based on waiting times and upper limits of the task time windows, the indicator is calculated.

(23) $$SN\left(𝓃\right)=Min\left\{\underset{𝒿}{\max}\left(W\left(𝒿,𝓃\right)\right),min\left\{\underset{𝒿}{\max}\left(0,B\left(𝒿\right)-𝓉\left(𝒿,𝓃\right)\right)\right\}\right\}$$

$W\left(𝒿,𝓃\right)$ is the waiting time of nurse 𝓃 at task 𝒿 after determining the floating of each independent nurse, and their arrival time to the first task as large as floating will be increased.

3. Rescheduling two

Once the starting time of the independent nurses’ tour has been corrected, the dependent nurses’ schedule will be automatically corrected. As the starting time of dependent nurses has not been changed in the rescheduling one, since the nurses are spending longer waiting, floating dependent nurses are calculated by rescheduling dependent nurses and adjusting the independent nurses’ scheduling, so it will take as long for dependent nurses to start their tour as their floating increases. Genetic mutations can result in infeasible chromosomes, which are volatile problem constraints during reproduction. The penalty function and repair mechanism described below are used to deal with these infeasible chromosomes.

#### 4.1.1. Initial Population Generation and Repair Mechanism

In this algorithm, the initial population is generated randomly. The genes in each chromosome are equivalent to tasks; start with the first gene and assign nurses randomly. It is important to note that assigning nurses to tasks requires several fundamental assumptions. To begin with, each task can only be handled by a group of nurses based on their abilities and patients’ preferences. As for the related tasks to a patient that should be done simultaneously by two individuals, only individuals who have both the qualifications and the desire to work together at the same time may be assigned these tasks, as well as those whose qualification degrees equal the required degree. Therefore, some of the generated chromosomes in the initial population generation process may not correspond to the problem assumptions. In order to resolve this issue, each chromosome generated will be repaired as follows:

- Step 1: For all tasks = 1: $N_{𝒿}$, if the assigned nurse to task 𝒿 does not qualify, choose one of the qualified nurses randomly and assign her to task 𝒿.
- Step 2: For each task that requires two people, determine the set of couples who are qualified and willing to cooperate, and the summation of their degrees is equal to the required degree. Go to step 3.
- Step 3: For each task that requires two people, choose one suitable couple randomly.

Three sections of the algorithm use the repair mechanism, following the generation of an initial population and the generation of children using crossover and mutation operators.

#### 4.1.2. Fitness Evaluation and the Penalty Function

A penalty function is used to handle infeasible solutions. During this process, infeasible chromosomes are not eliminated, but based on their deviation from the problem constraints, their penalty function will be worse, and their presence in the next generation will be reduced. These solutions should not be removed, because a small change made by a crossover or mutation may result in excellent objective functions for these chromosomes. Overall, the objective function of each chromosome equals the nurses’ total working time from the moment of departure from home to the moment of return. A number of constraints are not considered in the chromosome generation process, which could result in infeasible solutions. When a chromosome becomes infeasible, its objective function will increase by the following equation, where P is the penalty coefficient:

(24) $$OFV=P_{0}+P\times \left(P_{1}+P_{2}+P_{3}+P_{4}+P_{5}\right)$$

According to the chromosome design and decoding process, some constraints and assumptions are always established, while others will be held by repair mechanisms. However, in the five cases below, there may be a chromosome that becomes an infeasible case that, under this circumstance, chromosomes take the amount $P_{1}–P_{5}$. Consider a chromosome, if nurse 𝓃 starts task 𝒿, after the upper bound of the time window ($𝒷\left(𝒿\right)$), by the degree of deviation from $𝒷\left(𝒿\right)$, $pen1\left(𝒿,𝓃\right)$ is computed as below:

(25) $$pen1\left(𝒿,𝓃\right)=Max\left(0,𝓉\left(𝒿,𝓃\right)-𝒷\left(𝒿\right)\right);⟹P_{1}=\sum_{𝒿=1}^{N_{𝒿}}\sum_{𝓃=1}^{N_{𝓃}}pen1\left(𝒿,𝓃\right)$$

If task i is the prerequisite of task j, the time distance between the beginnings of those two tasks should be in the bound $\left[{𝒹}_{𝒾𝒿}^{min}.\;{𝒹}_{𝒾𝒿}^{max}\right]$. ${𝒹}_{𝒾𝒿}^{min}$ is always considered in the decoding process, but about ${𝒹}_{𝒾𝒿}^{max}$, equal to a deviation of it, penalty $pen1\left(𝒿,𝓃\right)$ is calculated as follows:

(26) $$pen2\left(𝒾,𝒿\right)=Max\left(0,\sum_{𝓃}𝓉\left(𝒿,𝓃\right)-\sum_{𝓃}𝓉\left(𝒾,𝓃\right)-{𝒹}_{𝒾𝒿}^{max}\right);⟹P_{2}=\sum_{𝒾=1}^{N_{𝒾}}\sum_{𝒿=1}^{N_{𝒿}}pen2\left(𝒾,𝒿\right)$$

Time that the nurse *n* waits to do task 𝒿 $\left(𝓌\left(𝒿,𝓃\right)\right)$ should be lower than ${𝓌}_{max}$. Otherwise, $pen3\left(𝒿,𝓃\right)$ is computed as below:

(27) $$pen3\left(𝒿,𝓃\right)=Max\left(0,𝓌\left(𝒿,𝓃\right)-{𝓌}_{max}\right);⟹P_{3}=\sum_{𝒿=1}^{N_{𝒿}}\sum_{𝓃=1}^{N_{𝓃}}pen3\left(𝒿,𝓃\right)$$

Each nurse should complete his/her task before a specific time $𝒷\left(𝓃\right)$ and goes to his/her destination. If the time of his/her arrival to the final destination ($𝓈\left(𝓃,𝓃\right)$) is more than $𝒷\left(𝓃\right)$, $pen3\left(𝓃\right)$ is computed as below:

(28) $$pen4\left(𝓃\right)=Max\left(0,𝓈\left(𝓃,𝓃\right)-𝒷\left(𝓃\right)\right);⟹P_{4}=\sum_{𝓃=1}^{N_{𝓃}}pen4\left(𝓃\right)$$

The time of each nurse’s activity (total time of servicing to different tasks), $𝓉𝓈\left(𝓃\right)$ should be lower than $D_{max}$. Otherwise, $pen5\left(𝓃\right)$ is computed as below:

(29) $$pen5\left(𝓃\right)=Max\left(0,𝓉𝓈\left(𝓃\right)-D_{max}\right);⟹P_{5}=\sum_{𝓃=1}^{N_{𝓃}}pen5\left(𝓃\right)$$

#### 4.1.3. Crossover and Mutation Operators

The crossover operators are used to generate children during the solution space search. The algorithm uses the Crossover one-cut-point operator and the Crossover two-cut-point operator to generate two children using two parents as follows:

Crossover procedure:

Step 1: Select two chromosomes $P_{1}=\left({𝓅}_{1}^{1},\ldots ,{𝓅}_{𝓃}^{1}\right)$ and $P_{2}=\left({𝓅}_{1}^{2},\ldots ,{𝓅}_{𝓃}^{2}\right)$ as parents. 

Step 2: Generate a random number r between 0 and 1; if 𝓇 < 0.5, go to step 3 (one-cut-point); otherwise, go to step 4 (two-cut-point).

Step 3: A position i between 1 to $N_{𝒿}$-1 is chosen randomly, and two offspring $O_{1}$ and $O_{2}$ are generated; $O_{1}=\left({𝓅}_{1}^{1},\ldots ,{𝓅}_{𝒾}^{1},{𝓅}_{𝒾+1}^{2},\ldots ,{𝓅}_{𝓃}^{2}\right)\;\mathrm{and}\;O_{2}=\left({𝓅}_{1}^{2},\ldots ,{𝓅}_{𝒾}^{2},{𝓅}_{𝒾+1}^{1},\ldots ,{𝓅}_{𝓃}^{1}\right).$

Step 4: Two positions: 𝒾 and 𝒿 between 1 and $N_{𝒿}$-1 are chosen randomly (𝒾 < 𝒿), and two offspring $O_{1}$ and $O_{2}$ are generated; $O_{1}=\left({𝓅}_{1}^{1},\ldots ,{𝓅}_{𝒾}^{1},{𝓅}_{𝒾+1}^{2},\ldots ,{𝓅}_{𝒿}^{2},{𝓅}_{𝒿+1}^{1},\ldots ,{𝓅}_{𝓃}^{1}\right)\;\mathrm{and}\;O_{2}=\left({𝓅}_{1}^{2},\ldots ,{𝓅}_{𝒾}^{2},{𝓅}_{𝒾+1}^{1},\ldots ,{𝓅}_{𝒿}^{1},{𝓅}_{𝒿+1}^{2},\ldots ,{𝓅}_{𝓃}^{2}\right).$.

**Figure 2:**
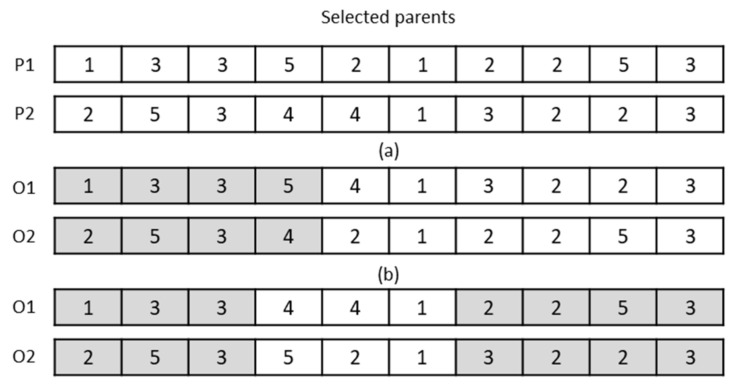
Crossover operators: (**a**) one-cut-point crossover with 𝒾 = 4 and (**b**) two-cut-point crossover with 𝒾 = 3 and 𝒿 = 6.

If $P_{1}$ and $P_{2}$ are two selected parents, $O_{1}$ and $O_{2}$ are two generated children, and a sample of the one-cut-point and two-cut-point operators is demonstrated in Figure 2. 

**Figure 3:**
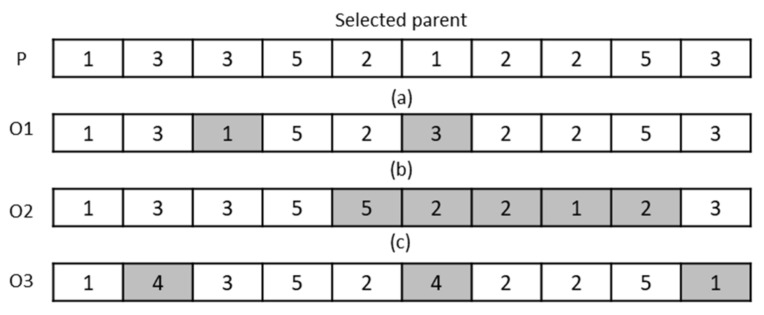
Mutation operators: (**a**) swap, (**b**) inversion, and (**c**) uniform.

Three types of mutation operators with equal probability are used to search for solution space and generate new solutions. By using the SWAP operator, two genes are randomly selected from one chromosome, and their amounts are supplanted with each other, producing a new solution point. A section of chromosome is randomly selected, and its genes amounts are sorted inversely in the inversion operator. Additionally, in a uniform operator, P𝓊% of the related chromosome genes are selected, and their amount will be supplanted by a random value between 1 and $N_{𝓃}$, where $N_{𝓃}$ is the number of nurses, and Pu is one of the algorithm parameters that can take every value between 0 and 1. For example, in Figure 3, if parent P is selected, $O_{1},O_{2},\;\mathrm{and}\;O_{3}\;$ are children that can be produced by each of three types of above mutations. As it is observed, each of these operators can create different changes in the parent chromosome and create a high capability to search the solution space in the regarded algorithm.

#### 4.1.4. Creating the Next Generation

Children are generated by crossovers and mutations, and then, some chromosomes are selected for the next generation. This can be accomplished through the use of two methods, elitism and the roulette wheel. All chromosomes are arranged according to their objective functions, and then, predetermined numbers of the best ones are selected for the next generation. The remaining members of the next generation are selected by using a roulette wheel selection that assigns a probability to each chromosome based on its fitness function. Therefore, chromosomes that have a higher fitness have a greater chance of being selected for the next generation. The following pseudocode can be used to represent the structure of the GA:
**Genetic Algorithm (GA)**  *The initialization of solutions and the required parameters of the algorithm.* *Set* φ=1; *Evaluate the fitness of solutions;*  *while the iteration*$\;\varphi \;\leq \varphi_{max}$*;*  * Generating new solutions using a crossover operator;* * Mutate some chromosomes randomly;* * Evaluate the objective values of the newly generated solutions;* * Generate the population for the next generation by selecting operator;*  φ=φ+1
*;*
 *end;*  *Return the most optimal solution.*      

### 4.2. PSO Algorithm

The PSO algorithm is a population-based random search method that was introduced by Eberhart and Kennedy [86], which originally was designed to solve continuous optimization problems. PSO is a widely used optimization algorithm for dealing with complex problems. There are several advantages to the PSO algorithm, including: a small number of algorithm parameters, the ability to parallelize it, and the ability to process it concurrently. It is an efficient global search algorithm. A thorough survey of the PSO algorithm with emphasis on the recent improvements can be found in [87]. This algorithm has also been proven to be efficient in routing and HHC problems in several papers [88].

The population and the potential solution are called swarm and particle, respectively. A particle has two major components; its speed and position, which are defined by the following equations:

(30) $${𝓋}_{𝒾}^{𝓀+1}=C(𝓌.{𝓋}_{𝒾}^{𝓀}+{𝒸}_{1}{𝓇}_{1}(pbest_{𝒾}-{𝓍}_{𝒾}^{𝓀})+{𝒸}_{2}{𝓇}_{2}(gbest-{𝓍}_{𝒾}^{𝓀}))$$

(31) $${𝓍}_{𝒾}^{𝓀+1}=round({𝓍}_{𝒾}^{𝓀}+{𝓋}_{𝒾}^{𝓀+1})$$

Here, ${𝓍}_{𝒾}^{𝓀}$ and ${𝓋}_{𝒾}^{𝓀}$ are the position and the speed of the 𝒾th particle in the 𝓀th iteration. The best particle 𝒾, which is obtained until iteration 𝓀, is shown by $pbest_{𝒾}$ and the best particle among all particles, which is obtained, is shown by $gbest,\;𝓌,{𝒸}_{1},\mathrm{and}\;{𝒸}_{2}$ are the inertia weight factor and acceleration coefficients, respectively. Additionally, ${𝓇}_{1}$ and ${𝓇}_{2}$ are two random numbers from (0,1). In the PSO approach, the move equations provide real, continuous values for the particle’s position, and since the solution space of this problem is discrete, the round function is used in Equation (31). Exploration requires a large inertia weight, while exploitation requires a smaller value. Therefore, to have a well-balanced inertia weight factor, the concept of a time-varying inertia weight factor was introduced in [89] and is given by the following expression:

(32) $$𝓌={𝓌}_{max}-({𝓌}_{max}-{𝓌}_{min})\times \frac{𝓀}{iter_{max}}$$

where ${𝓌}_{max}$ and ${𝓌}_{min}$ are the initial and final weight factors, $iter_{max}$ is the maximum number of iterations, and 𝓀 is the current iteration number. Usually, ${𝓌}_{max}=0.9,{𝓌}_{min}=0.4,$ and 𝓌 varies between ${𝓌}_{max}$ and ${𝓌}_{min}$. In Equation (30), C is the constriction factor. The acceleration of convergence of the algorithm is improved by C, [90] and [91], and is given by:

(33) $$C=\frac{2}{\left|2-\varphi -\sqrt{\varphi^{2}-4\times \varphi }\right|},\;\;\;\;\;\;\;\;\varphi ={𝒸}_{1}+{𝒸}_{2}>4$$

To avoid a PSO rush premature convergence, the concept of craziness was introduced in [92] by applying a certain probability and randomizing the velocity of some of the particles. The probability $\rho_{𝒸𝓇}$ is a function of inertia weight and defined as follows:

(34) $$\rho_{𝒸𝓇}={𝓌}_{min}-\exp(-\frac{{𝓌}^{𝓀}}{{𝓌}_{max}})$$

where ${𝓋}_{𝒿}^{𝓀}=rand\left(0,{𝓋}_{max}\right)$ if $\rho_{𝒸𝓇}\geq rand\left(0,1\right)$ and ${𝓋}_{𝒿}^{𝓀}\;$ otherwise. The following pseudocode can be used to represent the structure of the PSO:
**Particle Swarm Optimization (PSO)** *The initialization of solutions and the required parameters of the algorithm.* *Set* φ=1; *Evaluate the fitness of solutions;* ***while** the iteration*$\varphi \;\leq \varphi_{max}$*;* * Update velocity and position of the particles using*$V_{i}^{\varphi }=wV_{i}^{\varphi -1}+c_{1}r_{1}\left(pbest_{i}^{\varphi -1}-X_{i}^{\varphi -1}\right)+c_{2}r_{2}\left(gbest^{\varphi }-X_{i}^{\varphi -1}\right)$*and*$X_{i}^{\varphi }=X_{i}^{\varphi -1}+V_{i}^{\varphi };$ *  Evaluate the objective values of the particles;* *  Update the personal best positions;* *  Update the global best position;* φ=φ+1*;* ***end**;* *Return the most optimal solution.*

## 5. Numerical Examples

This section presents used instances in the first section. Afterwards, the parameters for the proposed algorithms are set, and the results are obtained using Cplex, and the proposed algorithms are compared. After that, the parameters of the model will be thoroughly analysed. The final section discusses managerial insight.

### 5.1. Case Study and Required Data

**Figure 4:**
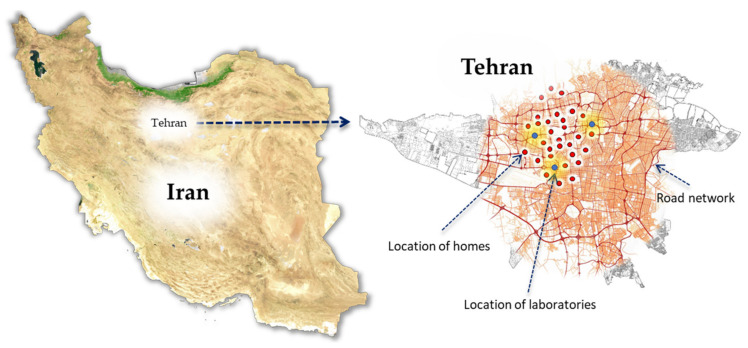
The location of case study.

A growing body of evidence supports the benefits of home health care. A wide variety of countries have experienced an increase in the demand for home health care services in recent years. Medical and social assistance needs are growing everywhere, but they are especially acute in countries where the average age of the population is rising and urbanization is accelerating. Tehran, Iran’s capital and largest city, has seen a number of medical service providers offering home health care services as a way to increase their patient base. In order to conduct this research, we collected information from these providers. Due to the sensitive nature of certain pieces of information, such as patient counts and addresses, these patients’ locations were randomly generated. This centre provides basic care at home for patients and elderly people, such as physical therapy exercises, medical tests, and monitoring medicines used by patients. Nursing services are provided by nurses according to their skill level in this centre. Some patients prefer to have a special nurse (based on the nurse’s gender or age) sent to them. In addition, some nurses do not prefer to work together because of cultural and social reasons. According to the literature, the above model takes into account the issues mentioned above, as well as some other aspects of the problem. Additionally, several local laboratories were considered as possible delivery points for the samples. The distance information was also retrieved from Google Maps. The location of the case study is shown in Figure 4.

According to the case, 30 problems were generated. There were three types of problems: small (instances: 1–14), medium (instances: 15–20), and large (instances: 21–30). Table 3 presents the features of each problem. Small-scale and medium-scale analyses of the model were performed using GAMS, while a large-scale analysis was carried out through metaheuristic algorithms. MATLAB was used to implement the algorithms, and a PC with a 1.80 GHz CPU and 6 GB of RAM was used to run them.

### 5.2. Parameters Setting

To tune the parameters of the proposed algorithms, an orthogonal experiment is conducted with a different set of parameters. In the GA, four parameters are selected as control factors: population size, crossover probability, mutation probability, and maximum iteration number, with three levels for each. For the PSO algorithm, four parameters with three levels are considered: maximum iteration number, swarm size, and the ${𝒸}_{1}\;\mathrm{and}\;{𝒸}_{2}$ coefficients.

**Figure 5:**
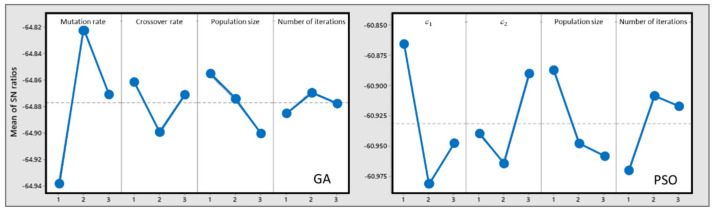
S/N ratio (smaller-the-better).

For both cases, the signal-to-noise (S/N) ratio is used to find the optimum level of controllable parameters. For the proposed problem, the smaller-the-better objective function in the Taguchi method is used. In Table 4, the levels of the parameters and their values are presented. The value of the signal-to-noise ratio is obtained by the following equation:

(35) $$S/N=-10\times log(\frac{\sum_{𝒾=1}^{𝓃}Y_{𝒾}^{2}}{𝓃})$$

where 𝓃 is the number of orthogonal arrays, and $Y_{𝒾}\;$ is the response value for the *i*th orthogonal array. The signal-to noise graphs are shown in Figure 5. 

Based on the Taguchi method, the best values and levels of the parameters are given in Table 5.

### 5.3. Results

A comparison of the solved instances using the proposed algorithms and Cplex is presented in Table 6. Since this problem is NP-hard, it can only be solved by Cplex software for small and medium instances. Cplex stops solving problems when the gap reaches 0.3 and the running time reaches 2, 5, and 10 for small, medium, and large problems, respectively. Since these algorithms are based on a random search of the solution space, there is no guarantee that the answers will be the same, regardless of their performance. Metaheuristics are run ten times for small and medium problems and five times for large problems. “The Best” column displays the best solution obtained in different algorithms, while “The Average” column displays the average objective function of each instance. This column represents the relative gap between the best-obtained solution of the proposed algorithms and the exact solution. A lower value indicates an algorithm with a greater efficiency.

(36) $$GAP=\frac{Best_{Algorithm}-Best_{CPLEX}}{Best_{CPLEX}}\times 100$$

**Figure 6:**
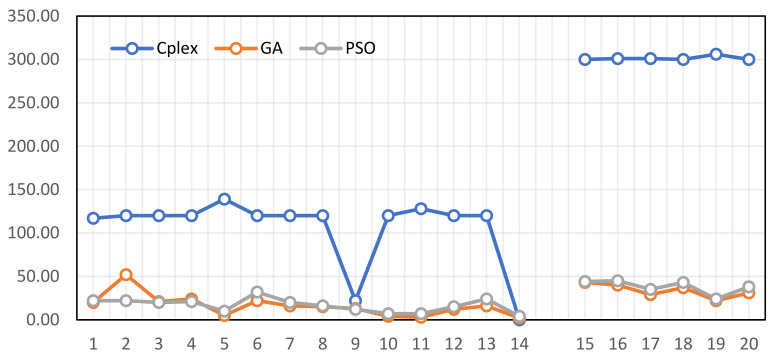
The comparison of Cplex and the proposed algorithms based on CPU time in small (1–14) and medium (15–20) instances.

According to Table 6, the proposed algorithms reach near-optimal solutions for the small and medium examples in a reasonable amount of time. Moreover, the proposed algorithms reached the solution in a reasonable amount of time for the large examples, where Cplex failed to reach the solution after 10 h. In almost every case, GA performed better than PSO. An illustration of the results can be found in Figure 6. It should be noted that, in some cases, the proposed algorithms provided better answers than Cplex. This is due to Cplex’s terminal stop condition to solve the problems.

## 6. Sensitivity Analysis

**Figure 7:**
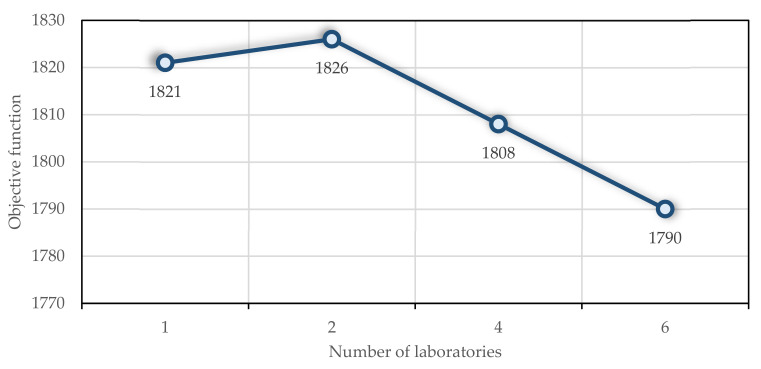
The impact of the number of laboratories on the objective function.

In order to evaluate the performance of the proposed model, a sensitivity analysis is conducted on a number of parameters. For this purpose, one instance is selected. The number of laboratories contracted by the home care centre is an important parameter. Due to the importance of maintaining social distancing, medical tests conducted at patients’ homes and transported to laboratories are some of the most important aspects of COVID-19. In this example, four patients must be sampled. Suppose this centre only has one laboratory contract, then increase the number of laboratories to two, four, six, and eight. This problem runs for two hours. For an increased number of laboratories, Figure 7 illustrates how the objective function behaves. As the number of contracted laboratories increases, the objective function behaviour decreases completely. Even after increasing the number of laboratories to eight, Cplex could not solve the problem in 10 h. To prevent computational problems associated with an increase in laboratories, an upper bound should be considered for laboratories that contract with home care centres. In the case of a considered instance with 60 tasks and only 4 requiring sampling, selecting more laboratories to send samples can significantly reduce nurses’ work time.

**Figure 8:**
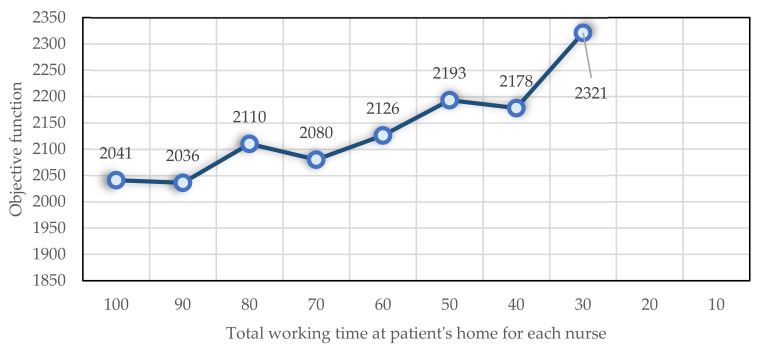
The impact of $D_{max}\;$ on the objective function.

**Figure 9:**
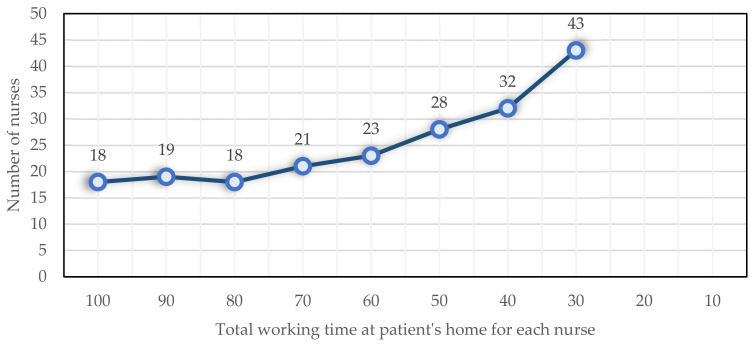
The impact of $D_{max}\;$ on the number of nurses.

As a next step, the total working time of each nurse in the patient’s home is changed, and its effect on the objective function and the number of nurses performing tasks is evaluated. At first, each nurse’s total working time in patients’ homes is 100 min, and then, it is decreased by 10 min each time. This problem becomes infeasible when the amount is between 10 and 20. As shown in Figure 8, the objective function increases when decreasing the total nursing time at patients’ homes. Additionally shown in Figure 9 is an approximately linear increase in the number of nurses employed. 

**Figure 10:**
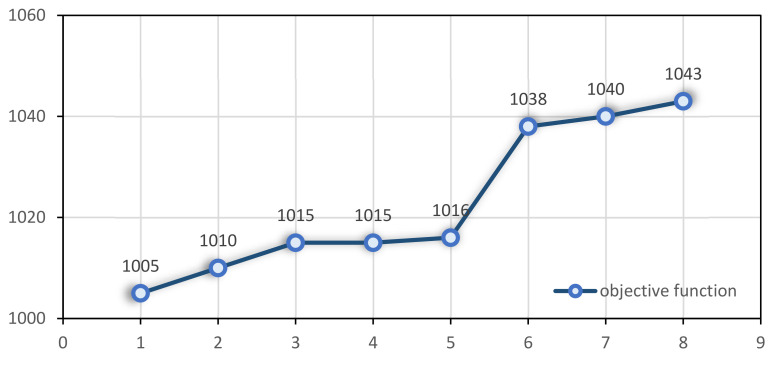
Impact of ${𝒷}_{𝓃}$ on the objective function.

In the following section, we examine the impact of the end time ${𝒷}_{𝓃}$ on the objective function. The nurses’ availability is first assumed to be 8 a.m. to 10 p.m.; then, it is decreased by 80 min at each stage. Cplex stops solving problems after 30 min of running time. In Figure 10, we can see how the objective function changes as ${𝒷}_{𝓃}$ decreases. The problem was not solved by Cplex within the specified time frame while reducing (case 9).

**Figure 11:**
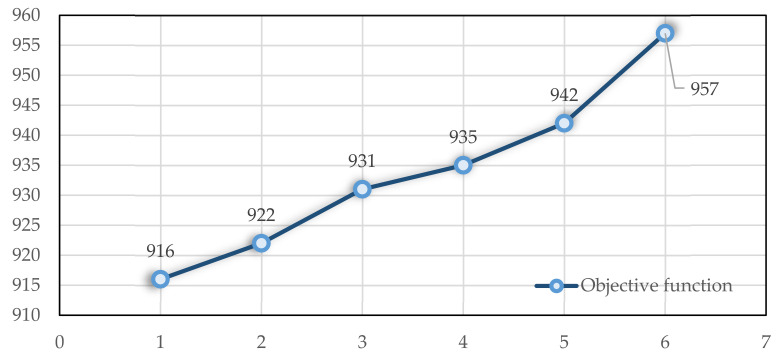
The impact of $A_{𝒿}\;\mathrm{and}\;B_{𝒿}$ on the objective function.

In the following section, we examine the effect of tasks’ time windows on the objective function. Every step adds 10 units to the initial value of the time window ($A_{𝒿}$) and subtracts 10 units from the final value ($B_{𝒿}$). Cplex stops solving problems after 30 min in all cases. Figure 11 shows how the objective function changes with a reduction in the task time windows. Figure 11 illustrates the general trend of changes in the objective function as the task time window is reduced. As the problem was relatively small, this parameter is expected to have a greater impact on large-scale problems. The last case (case 7) is unfeasible.

**Figure 12:**
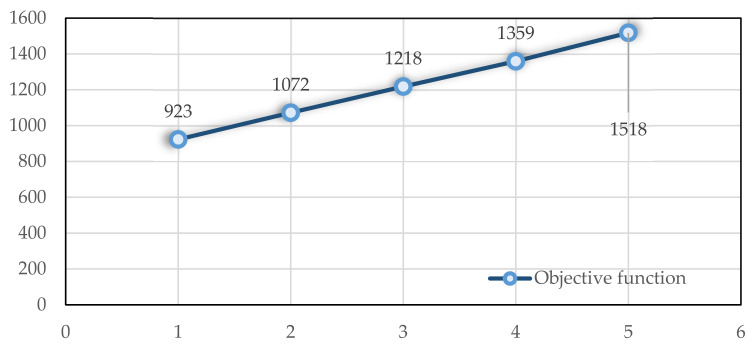
The impact of the duration of the tasks on the objective function.

Finally, we examine how the task duration affects the objective function. A total of 45 tasks are involved in the problem. At each step, each task takes 3 min longer. A terminal stop condition is reached after 30 min of running the Cplex to solve problems. As the task duration increases, Figure 12 shows how the objective function behaves.

## 7. Discussion and Managerial Insights

In the model discussed in this paper, nurses’ preferences for working with each other were examined. The delay in sending the sample to the laboratory can also affect the results of some laboratory tests, so this issue was considered a constraint in the model. In [13], the issue of sending the samples to the laboratory was examined. The model in this paper considered only one laboratory. Contrary to the previous paper, this study assumed that the home care centre could contract with several laboratories and investigated the effect of this parameter on the objective function. The authors of [66] presented a bi-objective model for minimizing the total service time and total cost. In contrast to the present study, the laboratory and hospital were the starting and ending points. Furthermore, the sample delivery time to the laboratory was not taken into account. 

We examined the effect of changing the main parameters of the model on variables and the objective function in the previous section. As a result of the analysis, the following economic and managerial insights are presented:

- As shown in Figure 6, planners are advised to reduce nurses’ working time by increasing the number of laboratories during shortages of nurses or when epidemics such as COVID-19 occur. It is also better to consider an upper bound for laboratories that are contracted with home health care centres to prevent computational problems.
- There is an inverse relationship between $D_{max}$ (the total working time of each nurse) and the number of nurses used. When $D_{max}$ decreases, more nurses are used. It is possible to control the number of nurses used, as well as the value of the objective function, by selecting the appropriate value for $D_{max}$. Management is also advised to increase nurses’ working time as much as possible to hire fewer nurses and reduce costs.
- According to Figure 7 and Figure 9, if the ending time of the nurses’ time window (${𝒷}_{𝓃}$) is reduced for some nurses, the total working time for each nurse at a patient’s home can be increased to reduce the slope of the total costs increase.
- The availability time of nurses directly impacts the value of the objective function (especially for large-scale instances), so it is better to contract with nurses who are available for longer hours.
- Considering Figure 10 and the effect of the time windows on the objective function, it is suggested that management consider the time window constraint as a soft constraint for some tasks.
- In Figure 11, management is advised to have a forecast for the increase in nurses if the duration of tasks increases for various reasons.

## 8. Conclusions

A new mathematical formulation of the home health care routing and scheduling problem was presented in this paper. A nurse’s preference and skill level were considered in synchronizing visits. In addition, the home care centre was assumed to have contracts with several laboratories for the transfer of samples. Analyses were conducted on the consequences of this policy. This study also considered the number of laboratories. Based on the sensitivity analyses, it was found that the objective function decreases as the number of laboratories increases. Additionally, laboratory delivery times were taken into account. Specified tests must reach the lab within a certain period of time in order to be valid for testing. In order to solve large instances, a new three-stage GA and a PSO algorithm were proposed. The proposed algorithms were shown to be effective based on the results. Based on the obtained results, it is possible to conclude that the quality matrix and the complexity of the instances are related. It implies that, in addition to the number of instances (number of nurses, number of tasks, etc.), the quality matrix has an undeniable effect on the problem’s complexity and, consequently, its solution time. The impact of the quality matrix on the problem is associated with difficulties and has received less attention in the literature, despite its importance. In addition, the number of laboratories with which the HHC centre has a contract reduced nurses’ working times. Governments are committed to improving health indicators. A significant portion of the government budget is devoted to health care. Consequently, health sector costs can be reduced through proper management. HHC can serve as an excellent alternative to health centre-based services in this regard.

It is possible to expand this work in many directions.

- Laboratory locations and numbers can be considered as decision variables in the model. Furthermore, the quality matrix has a significant impact on the model’s complexity, which is rarely discussed in the literature, in addition to the problem’s dimensions (number of tasks, number of nurses, and number of synchronization tasks).
- Further, exploring priority queueing systems for determining the optimal nurses [93,94] integrated with HHP would be an intriguing future research area due to the growing demand for nurses with various conditions.
- Additionally, consider the legal restrictions and uncertainties associated with exceptions and permanent changes. In addition, taking into account the cultural challenges, payment methods, and an overall lack of satisfaction with the work is important.
- Moreover, the types of vehicles used for transporting samples and nurses have not been investigated in this paper but can be examined further in future studies.
- The use of machine learning to predict HHP demands could be an interesting research topic in the future [95].

## Figures and Tables

**Table 2:** Summarizes the notations of the model.

Sets	
N	Set of nurses
J	Set of tasks
$J^{pre}$	Set of tasks with precedence
O	Set of starting location of nurses
L	Set of ending location of nurses
$J^{Lab}\subseteq J$	Set of tasks requiring sampling
Lab	Set of laboratories
**Parameters**	
${𝓆}_{𝒾𝓃}$	Equals 1 if nurse 𝓃 is qualified to perform task 𝒾
${𝒹}_{𝒾𝒿}^{min}$	The maximum time distance between tasks (𝒾 and 𝒿) with precedence
${𝒹}_{𝒾𝒿}^{max}$	The minimum time distance between tasks (𝒾 and 𝒿) with precedence
$\mu_{𝒾𝒿}$	Equals 1 if tasks 𝒿 must be performed after task 𝒾
$𝓰{𝓇}_{𝓃}$	Grade of nurse 𝒿
$𝓰{𝓇}_{𝒿}$	Grade of task $𝒿\in J^{pre}$
$𝓅{𝓇}_{𝓂𝓃}$	Equals 1 if two nurses’ 𝓂 and 𝓃 want to work together
${𝒹}_{𝒾}$	Duration of task 𝒾
$D_{max}$	Maximum working time (not including travelling time) for each nurse
$W_{max}$	Maximum waiting time at each task
${𝓉}_{𝒿}^{max}$	The maximum time for sending samples related to task 𝒿 to the laboratory
${𝓉}_{𝒾𝒿}$	Travelling time between $\left(𝒾,\;𝒿\right)$
$\left[{𝒶}_{𝓃},{𝒷}_{𝓃}\right]$	The time window for nurse 𝓃
$\left[A_{𝒿},B_{𝒿}\right]$	The time window for task 𝒿
M	Big number
**Variables**	
${𝓍}_{𝒾𝒿𝓃}$	Equals 1 if nurse 𝓃 moves from 𝒾 to 𝒿
${𝓈}_{𝒿}$	Starting time of task 𝒿
${𝓌}_{𝒿}$	Waiting time at task 𝒿

**Table 3:** Characteristics of the instances.

Instance	Number of Tasks	Number of Nurses	Number of Tasks with Precedence	Number of Tasks Require Sampling	Number of Laboratories	Instance	Number of Tasks	Number of Nurses	Number of Tasks with Precedence	Number of Tasks Require Sampling	Number of Laboratories
1	18	30	10	2	2	16	66	50	6	4	4
2	74	60	4	4	4	17	70	50	6	4	4
3	60	20	4	4	4	18	70	45	6	4	4
4	56	30	6	4	4	19	56	35	6	4	1
5	20	20	10	2	2	20	70	30	6	4	4
6	78	50	4	4	4	21	96	50	6	4	4
7	62	20	4	4	2	22	78	60	8	4	4
8	60	25	6	4	4	23	76	60	6	4	2
9	46	20	6	4	1	24	65	60	20	5	1
10	20	10	10	2	2	25	56	50	20	5	1
11	20	8	10	2	2	26	65	60	20	5	1
12	64	15	4	4	4	27	70	50	6	4	4
13	60	20	6	4	4	28	82	60	8	4	4
14	11	8	4	2	2	29	66	55	20	5	1
15	66	55	6	4	4	30	76	60	20	5	1

**Table 4:** Algorithm’s parameter settings.

Algorithm	Parameter	Level 1	Level 2	Level 3
GA	number of iterations	1400	1600	1800
	Mutation rate	0.05	0.07	0.09
	Crossover rate	0.5	0.7	0.9
PSO	Population size	40	50	60
	number of iterations	4000	5000	6000
	${𝒸}_{1}$	2.05	2.1	2.2
	${𝒸}_{2}$	2.05	2.1	2.2
	Swarm size	160	120	140

**Table 5:** The tuned algorithms’ parameters.

Algorithm	Parameter	Best Value
GA	Number of iterations	1600
	Mutation rate	0.07
	Crossover rate	0.5
	Population size	40
PSO	number of iterations	5000
	${𝒸}_{1}$	2.05
	${𝒸}_{2}$	2.2
	Swarm size	120

**Table 6:** Comparison of the solutions obtained by the exact solution, GA, and PSO.

	CPLEX		GA				PSO			
Test Problem	OF	Time (h: min: s)	The Best	Time	GAP (%)	Average	The Best	Time	GAP (%)	Average
1	1001	1:57:33	1015	0:20:31	1.40%	1021	1071	0:22:33	6.99%	1106
2	2021	2:00:43	2041	0:52:25	0.99%	2052	2125	0:22:41	5.15%	2179
3	1645	2:00:04	1661	0:21:51	0.97%	1679	1710	0:20:18	3.95%	1728
4	1449	2:00:19	1475	0:24:39	1.79%	1483	1510	0:21:50	4.21%	1528
5	927	2:19:45	926	0:05:22	−0.11%	931	945	0:10:54	1.94%	965
6	1955	2:00:09	1915	0:22:40	−2.05%	1926	1942	0:32:07	−0.66%	1960
7	1481	2:00:13	1496	0:16:03	1.01%	1502	1501	0:20:24	1.35%	1512
8	1318	2:00:12	1320	0:15:46	0.15%	1324	1326	0:16:03	0.61%	1328
9	1037	0:22:46	1025	0:13:42	−1.16%	1030	1032	0:12:54	−0.48%	1042
10	969	2:00:11	969	0:04:26	0.00%	978	978	0:07:06	0.93%	986
11	371	2:08:46	371	0:03:55	0.00%	375	380	0:07:09	2.43%	389
12	1684	2:00:25	1692	0:12:08	0.48%	1698	1696	0:15:42	0.71%	1705
13	1465	2:00:15	1465	0:16:40	0.00%	1472	1475	0:24:42	0.68%	1480
14	324	0:00:40	324	0:02:32	0.00%	326	324	0:04:38	0.00%	328
15	2313	5:00:37	2354	0:43:03	1.77%	2381	2416	0:44:45	4.45%	2458
16	2449	5:01:35	2511	0:40:46	2.53%	2534	2580	0:45:15	5.35%	2611
17	2318	5:01:49	2332	0:29:48	0.60%	2366	2341	0:35:07	0.99%	2348
18	2534	5:00:27	2534	0:37:58	0.00%	2538	2561	0:43:09	1.07%	2582
19	1673	5:06:02	1682	0:22:20	0.54%	1686	1690	0:24:04	1.02%	1697
20	2294	5:00:17	2308	0:31:08	0.61%	2316	2322	0:38:42	1.22%	2331
21	-	10:00:05	-	1:53:00	-	2643	-	2:04:22	-	2710
22	-	10:01:02	-	1:23:26	-	2587	-	1:44:25	-	2612
23	-	10:00:45	-	1:16:59	-	2320	-	1:23:32	-	2373
24	-	10:01:20	-	1:03:27	-	1627	-	1:10:08	-	1684
25	-	10:04:36	-	0:46:30	-	1588	-	0:46:56	-	1614
26	-	10:00:03	-	1:01:17	-	1774	-	1:10:36	-	1807
27	-	10:07:04		0:34:57	-	2880	-	0:40:38	-	2928
28	-	10:00:02		0:46:41	-	2526	-	1:03:54	-	2541
29	-	10:00:07		0:51:17	-	1610	-	0:46:27	-	1621
30	-	10:00:14		1:08:00	-	1836	-	0:53:47	-	1853

## Data Availability

The data used in the study are available with the authors and can be shared upon reasonable request.
